# Evaluating medical students’ knowledge of patient-reported outcomes and the impact of curriculum intervention in consecutive cohorts

**DOI:** 10.1186/s41687-023-00670-z

**Published:** 2023-12-13

**Authors:** Samuel A. Florentino, Suzanne B. Karan, Gabriel Ramirez, Judith F. Baumhauer

**Affiliations:** University of Rochester School of Medicine & Dentistry, 601 Elmwood Ave, Box 192, Rochester, NY 14642 United States

## Abstract

**Background:**

Patient-reported outcomes (PROs) collection and utilization improves patient-provider communication, symptom reporting, and patient satisfaction. Despite their significance, the science and utility of PROs are not part of required curriculum in medical education. The authors describe the results of a survey distributed to medical students evaluating their experience, knowledge, and perceptions of PROs, report on outcomes of the impact of formal PRO education on medical student knowledge, and describe strategies to foster the spread of PRO education into other programs.

**Methods:**

The authors developed and distributed a 20-question web-based survey distributed to medical students at two U.S. medical schools to evaluate students’ experience, knowledge, and perceptions of PROs. To compare medical students’ knowledge in their pre-clinical years (M1-M2) to those in their clinical years (M3-M4), the authors calculated odds ratios and determined significance determined using chi-squared tests. To determine the utility of formal education on medical students’ knowledge of PROs, the authors invited 4th year medical students at a single institution to participate in a survey before and two weeks after receiving formal PRO education as part of the medical school curriculum, spanning three years.

**Results:**

137 (15%) medical students responded to the initial survey. Respondents’ knowledge of PROs was low and did not differ when comparing pre-clinical to clinical years in school. Less than 10% had received education on PROs and only 16% felt prepared to use PROs in patient care. Respondents demonstrated positive attitudes towards PROs, with 84% expressing interest in learning about PROs. In the second phase education cohort of 231 (77% response rate) 4th -year medical students over three years, formal education improved correct response rates to PRO questions. After education, 90% (121/134) agreed PROs are an important component of high-quality care.

**Conclusions:**

This study identifies a gap in knowledge about PROs among medical students irrespective of year in training. It also shows that structured education may help fill the PRO knowledge gap, potentially providing future clinicians with the skills to implement PROs into clinical practice, aligning with the broader shift towards patient-centric evidence-based healthcare practices.

**Supplementary Information:**

The online version contains supplementary material available at 10.1186/s41687-023-00670-z.

## Background

Patient-reported outcomes (PROs) are any report of a the status of a patient’s health condition, health behavior, or experience with healthcare that comes directly from the patient, without interpretation of the patient’s response by a clinician or anyone else, and therefore directly reflect the voice of the patient [1]. While simple question relating to symptoms and well-being have always been a part of medicine, more recently validated PRO measures been developed. In this article “PROs” refers to this latter type. PROs are being increasingly utilized in clinical practice and provide clinicians with efficient and relevant information regarding how the patient is feeling and functioning in the context of their health status. The clinical utility of PROs is increasingly evident as they are now incorporated in clinical guidelines and board certification [2]. Current evidence suggests that routinely collected PROs improve patient-provider communication, symptom reporting, increase patient satisfaction and engagement, and lead to changes in patient treatment, decision-making, and management [3–5]. However, providing sufficient training and education for both patients and clinicians is an important step in linking clinician action and intended improved patient outcomes. The ability to look at aggregate data over time for disease conditions and to assess impact on treatment has been incorporated into electronic health system records to be used for both shared decision-making and to assess value in healthcare [4]. Despite the increasing importance of PROs in clinical care, there appears to be a knowledge gap regarding the understanding of these important patient-centered tools and their clinical use to aid in treatment decision-making and align patient’s expectations with treatments. Upstream education, at the medical student level, could help with downstream care of patients.

Currently, the utilization of PROs to improve patient care is becoming increasingly routine [4]. However, neither the Liaison Committee on Medical Education (LCME) accreditation standards nor the Accreditation Council for Graduate Medical Education (ACGME) includes PRO instruction as part of the medical school or residency education requirements. As a result, medical school graduates could be unprepared to use them when they start their residency or fellowship. This will serve as a barrier to patient-centered high-quality care. We developed, rigorously pre-tested, and distributed a survey to evaluate contemporary knowledge of PROs among students in U.S. medical schools. We also assessed medical students’ knowledge before and after receiving formal education of PROs over a three-year period.

## Methods

This study was reviewed by the University of Rochester Institutional Review Board and determined to be exempt (STUDY00006278).

### Aim 1: survey of medical students

In the first part of our study, we assessed medical students’ knowledge, experience, and perceptions of PROs through a survey.

### Survey

Survey development began with an extensive PubMed literature search. Key search terms included patient-reported outcome, patient-reported outcome measurement, information system, PROMIS, patient-reported, and self-reported.

Following the literature review, we developed a 20-question survey.

### Pre-testing of the survey to improve validity

Following the development of the primary survey questions, we evaluated and revised the language and topics to enhance clarity and comprehensiveness. The survey was uploaded to an online (REDCAP) platform and pretested with five medical students of varying levels of training via cognitive interviewing. The cognitive interviews were conducted using the flexible verbal probing method and adaptive procedure [6]. Interviewees’ responses were compared to identify inconsistencies in the interpretation of the survey questions. Survey questions and answer choices were then revised to assure completeness, clarity, and inclusiveness of the survey questions and answer choices. Pilot testing of the revised survey was performed with ten medical students including students from each year (M1-M4). The pilot survey was distributed through the same online platform used for final distribution to replicate the process [7, 8]. Participants were given seven days to respond. The pilot testing demonstrated that the implementation of the survey was feasible, including both distribution and data collection.

### Survey distribution

All LCME accredited medical schools in the United States were contacted between August and October of 2021, with a request to distribute the survey to their medical students (M1-M4 and MD-PhD students). A letter template for email distribution to students was provided. Invitations, sent directly from our research team, emphasized that survey participation was voluntary, anonymous, and confidential. Within the body of the email was a disclaimer that the survey was voluntary and would not influence grading, and a request not to use educational materials while answering the survey and to take the survey only once. An initial email containing a link to the survey was available to be sent by each participating institution and a reminder email was subsequently sent to students one and two weeks later.

### Survey questions

Respondents’ familiarity with PROs was gathered based on the responses to four survey questions (questions 1, 2, 3, and 4; Supplemental Digital Appendix 1). Medical students’ knowledge of PROs was assessed with seven questions (questions 5 through 11) and were categorized as correct or incorrect. These knowledge questions included content area covering PRO definition (question 5), application (question 6), patient engagement (question 7), Centers for Medicare & Medicaid Services (CMS) utilization (question 8), implementation (question 9), reporting (question 10), and privacy (question 11). Correct responses were determined according to citable evidence-based recommendations (Table 1). For questions where the answer choice included “select all”, responses were counted as correct if respondents selected all correct responses and did not select any incorrect responses.

**Table 1:** Areas of PRO knowledge evaluated with corresponding reference

Knowledge	Origin of correct response
Definition of PRO	NQF, CMS MMS, 2013 Weldring, 2018 Gensheimer
Case vignette identification of PROs	NQF, CMS MMS, 2013 Weldring, 2018 Gensheimer
Shared decision making and patient engagement with PROs	NQF, 2020 Price, 2018 Gensheimer
Centers for Medicare & Medicaid Services use of PROs	2020 cm Blueprint
Implementation of PROs	NQF, CMS MMS, 2018 Gensheimer
PRO reporting	2018 Cella, 2021 Lapin,
PRO shared decision making, privacy	NQF, 2018 Gensheimer

PRO = Patient Reported Outcome

NQF = National Quality Forum

CMS MMS = Centers for Medicare & Medicaid Services Measured Management System

### Aim 2: formal education

In the second part of our study, we sought to evaluate the impact of formal education on medical students’ knowledge of PROs. To this end, we compared the responses of medical students before and after receiving formal education at the University of Rochester. A 50-minute educational lecture on PROs, with a supplemental slideshow, was delivered to 4th -year medical students by one international clinician scientist PRO expert (J.F.B). The objective of the lecture was to introduce the concept of PROs, explain measurement methods, interpretation, and illustrate PRO use in the clinics. The lecture was delivered in person to three chronologic 4th-year classes, with the lectures occurring in February of 2021 2022, and 2023.

### Modification of survey for pre- and post-education assessment

A shortened survey using key questions from the previously described survey that corresponded with the learning objectives was distributed to medical students before and after the educational lecture. Invitations stated the completion of the survey was voluntary, anonymous, confidential, and would not affect course grading.

The four-question survey was distributed to the 4th-year medical students by email one day before the lecture and consisted of questions inquiring about students’ familiarity with (question 1) and knowledge of (questions 2, 3, and 4) PROs. We administered the post-education survey via email two weeks later to reduce recency effect. The post-education assessment included the same four questions as the pre-education survey, with a fifth question inquiring about students’ perception of PROs and their impact regarding patient care.

### Statistics

Rates are shown as a percentage of correct responses for questions assessing knowledge of PROs. To compare medical students’ knowledge in their pre-clinical years (M1-M2) to those in their clinical years (M3-M4), we calculated odds ratios and determined significance using chi-squared tests, using responses to key questions from the survey. The anonymity of the results of formal education prevented a direct contrast between the pre-education and post-education survey responses, and therefore the analysis was restricted to separate descriptive statistics of the pre- and post-education survey responses. The analysis was performed using Stata-MP 16.1 for Windows (Stata Corp, College Station, Texas). Study data were collected and managed using REDCap electronic data capture tools hosted at the University of Rochester.

## Results

### Aim 1: survey of U.S. medical students

Two medical schools distributed the initial survey to their medical students (University of Rochester and Geisinger Commonwealth). In total, 137 medical students responded to the survey. The total response rate of medical students who received the survey was 15% (137/936). The demographics of medical students who responded to the survey are defined in Table 2. The 34 students that did not respond to demographic information did not have their responses included in the comparative analysis of students by their year in school, while their other responses were included in reporting.

**Table 2:** Demographics of Respondents

**Year in School (N = 103)**	Number of responses (%)
1st year	26 (25.2)
2nd year	35 (34.0)
3rd year	20 (19.4)
4th year	18 (17.5)
Other (MD/PhD)	4 (4.9)
**Months of Clinical Rotations** ** (N = 103)**	
None	50 (48.5)
> 3 months	23 (22.3)
3 to 6 months	6 (5.8)
> 6 to < 12 months	7 (6.8)
> 12 months	17 (16.5)
**Gender Identity** ** (N = 101)**	
Female	61 (60.4)
Male	38 (37.6)
Non-binary/third gender	0
Transgender female	0
Transgender male	0
Preferred not to say	1(1.0)
Other	1 (1.0)

Percentages were calculated out of the total number of respondents for a given question, N

### Students’ experience with PROs

Approximately 58% (79/137) of responding students reported having knowledge of PROs. Fewer than 8% (10/137) had ever received formal education on PROs, while 23% (32/137) reported using PROs as a student, researcher, or employee. Further, 9% (13/137) had observed a provider utilizing a PRO in patient care. Only 16% of respondents felt prepared to utilize PROs in a patient care setting.

### Students’ knowledge of PROs

Fifty-five percent (59/108) of respondents correctly identified the definition of a PRO; however, fewer than 5% (5/107) correctly identified PRO elements within a case vignette. Approximately one-fifth of respondents (21%, 22/105) correctly responded when asked about the purpose of the recommendations by the Centers for Medicare & Medicaid Services (CMS) to incorporate PROs into patient care. A third (33%, 35/105) of medical students correctly identified the necessary features for the incorporation of PROs into daily practice. Less than 71% of respondents correctly answered when asked about specific PRO topics, including shared decision-making (66%, 69/105, question 7), reporting (64%, 66/103, question 10), and privacy (70%, 72/103, question 11).

### Students perception of PROs

**Fig. 1:**
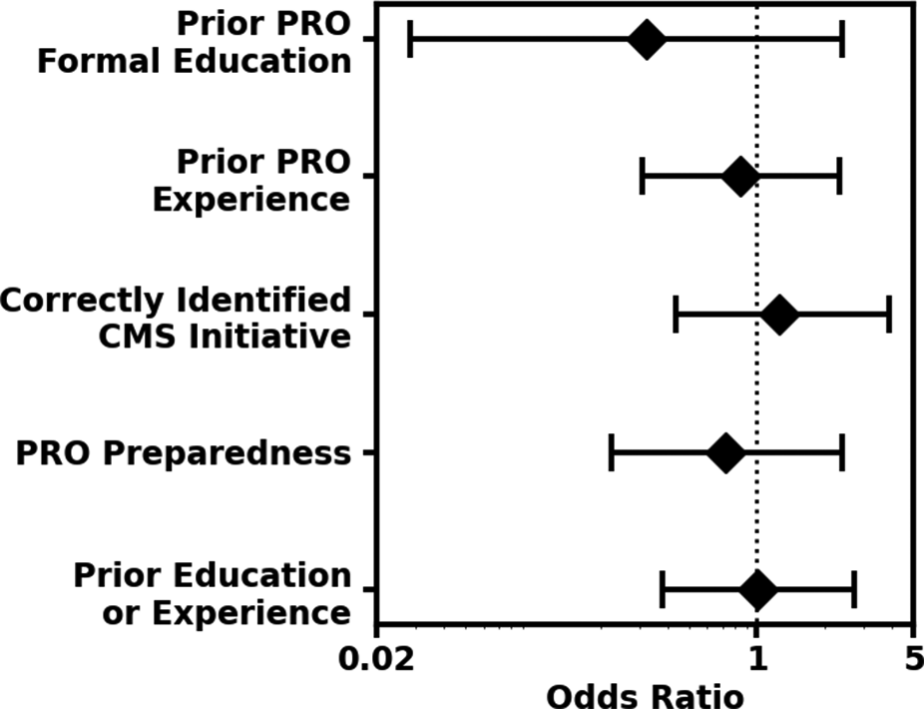
Odds ratios demonstrating that medical student knowledge, experience, and perception did not significantly differ when comparing those in their pre-clinical years (M1-M2) to those students in their clinical years (M3-M4). Survey questions used are 2 (education), 3 (experience), 8 (CMS), 13 (preparedness) in supplemental digital appendix 1. Prior PRO Education Odds ratio [OR]: 0.32; 95% confidence interval [CI]: 0.03, 2.40; p-value: 0.18). Prior PRO experience (OR: 0.84; CI: 0.31, 2.336; p-value: 0.71). Correctly identified Centers for Medicare and Medicaid Services (CMS) initiative (OR: 1.27; CI: 0.44, 3.89; p-value: 0.64). PRO preparedness (OR: 1.12 CI: 0.45, 2.81; p-value: 0.80). Prior education or experience (OR: 1.00 CI: 0.38, 2.74; p-value: >0.99)

Respondents demonstrated positive attitudes toward PROs. Greater than three-quarters of responding medical students (76%, 78/102) agreed PROs are important in delivering high-quality patient care with 66% (67/102) planning to utilize PROs in future practice. Most medical students (84%, 86/102) were interested in learning about PROs. Medical student responses did not significantly differ by year in training when examining key PRO concepts, prior education or experience, or perceived preparedness to utilize PROs (Fig. 1).

### Aim 2: formal education

A total of 301 4th-year medical students attended the formal education lecture on PROs over three years.

### Pre-education

77% (231/301) of 4th-year medical students responded to the pre-educational survey. The results of students’ responses to PRO knowledge questions are summarized in Table 3.

**Table 3:** Patient Reported Outcome (PRO) knowledge before and two weeks after a single educational lecture implemented into 4th year medical student curriculum

	Before Education (n/N, %)	Two Weeks After Education (n/N, %)
Differentiate PROs from other outcome measures	140/231 (60.6%)	113/141 (80.1%)
Define a PRO	8/231 (3.5%)	34/141 (24.1%)
Case vignette identification of PROs	30/231 (13.0%)	30/141 (21.3%)

n = Number of correct responses

N = Total number of responses

Percentages were calculated from the number of correct responses out of the total number of responses for a given question

### Post-education

Nearly half of students (47%, 141/301) who attended the educational session responded to the post-lecture survey. Overall, there was an increase in correct response rates noted on all questions. Additionally, most medical students (90%, 121/134) agreed PROs are an important component of providing high-quality care while the remaining students were unsure (10%, 13/134).

## Discussion

This report is the first to evaluate the knowledge gap among medical students regarding the interpretation and use of PROs. This research also provides insight into medical students’ attitudes regarding PROs. The initial survey results from two different medical schools revealed limited knowledge of PRO assessment and utilization among medical students. Important findings include: (1) low levels of knowledge of PROs; (2) a desire to receive education on PROs; (3) unpreparedness to utilize PROs in a clinical setting; (4) overall positive attitudes towards PROs. Education, knowledge, and attitudes did not vary by year-in-training. In a three-year assessment at a single institution, incorporating a 50-minute formal educational session into medical education curriculum improved correct response rates in fourth-year medical students’ knowledge of PROs.

In total, the identified deficiencies in PRO knowledge support the need to implement PROs into medical education curriculum and a lecture can help fill that gap. Accordingly, incorporating PRO education into medical education could be helpful and supports the initiative of the American Medical Association (AMA) for medical schools to adopt health systems science as the third pillar of medical education [9, 10]. PROs are an important aspect of health systems science as they provide crucial insight into patients’ perspectives of their health and healthcare experience. More broadly, PRO collection and use can help to improve the quality and value of care at the individual patient level and when assessing population health initiatives [3–5, 11–14]. The inclusion and melioration of PRO education in the medical school curriculum seems necessary to improve the students’ ability to deliver high-quality care as future clinicians. The diverse utility and breadth of PROs can help align healthcare delivery with the needs and preferences of patients, leading to better outcomes while placing the patient front and center in the equation. Published clinical examples of the utility of PROs to improve patient care has been demonstrated in numerous fields and specialties. For example, Basch et al. found collecting PROs outside of regularly scheduled cancer treatment visits, such as at home or when the patient feels it is important for the clinician to know, positively impacted the health of the patients with fewer emergency room visits and improved mortality rates [5].

With any survey study, there are limitations. The findings of the first part of our study, the survey implementation amongst U.S. Medical Schools, are based on respondents from two medical schools with an overall low response rate (15%), therefore generalization to medical students at other institutions may be limited. PRO education is not currently required in contemporary medical education by the Liaison Committee on Medical Education (LCME), and it appears to be a lower priority of medical schools when constructing medical education curricula at this time. Additionally, our primary survey has a low overall response rate (15%) and is subject to non-response bias. Similarly, the post-education survey had a low response rate (47%) compared to the pre-education survey (77%), and therefore a greater likelihood of non-response bias. Therefore, cautious interpretation is necessary regarding the effectiveness of the single educational session. The participatory nature of surveys may contribute to non-response bias, in which medical students choose not to complete surveys that are not of interest or in which they lack the knowledge to complete. Other factors contributing to a low response include failed delivery, in which emails distributed to medical students were not viewed, and omission, in which students forgot to complete the survey. Also, social desirability bias may have led students to respond to questions based on social expectations, rather than true attitudes towards PROs [15]. A major strength of this study is the utilization of a survey that underwent extensive development, rigorous pre-testing and piloting.

Regarding formal education of PROs in a single institution, response rates of pre- and post-educational surveys were satisfactory (76% and 47%, respectively), and most students (> 90%) agreed that PROs are important in providing high-quality care. Another strength of our study is the incorporation of a low-cost, low time commitment education initiative which provides genuine and real-world insight into the feasibility of its implementation at other institutions, while the increase in correct response rates seen in three consecutive years supports its efficacy.

We acknowledge this educational experience alone is insufficient for students to master the skills necessary to utilize PROs as they transition to residency and beyond. Therefore, next steps for medical education programs will be to include PRO education into the curriculum, provide opportunities for students to practice the use of PROs for symptom monitoring and choosing treatment plans during skills sessions and clinical rotations. Subsequent experience on clinical rotations, with viewing and using the data with clinicians, will be needed to reinforce and expand future clinician’s knowledge and ability to utilize PROs to better understand patients’ experience of illness and address their needs. Incorporating PRO education into medical education curriculum will likely be more effective at institutions that have already instituted PROs as a standard in patient care. For example, at the University of Rochester Medical Center, many departments and divisions have incorporated PROs as the standard of care to assess how a patient is feeling and functioning, with the PROMIS assessments being the greatest number of tools collected. As of June 2023, over 325,000 unique patients have completed PROs providing clinicians with added information as well as the ability to look at aggregate data over time for focusing on disease conditions or treatment plans [4]. As a result, students can learn about PROs, apply this tool in patient care, and ultimately feel prepared to utilize them. This approach would foster students’ confidence in their ability to utilize PROs, support the initiatives of both HSS and CMS to improve the quality of care by highlighting the voices of patients, and assist future physicians in adjusting to the value-based payment reform in the U.S. healthcare system [16, 17].

## Conclusions

In conclusion, this study identifies a knowledge gap among medical students regarding the interpretation and utilization of PROs. Given the established advantages of integrating PROs into care, the inclusion of formal PRO education within medical curricula emerges as a promising approach to bridge this gap. This step has the potential to equip upcoming clinicians with essential skills for PRO implementation in clinical practice. By addressing this knowledge gap through structured education, the study not only provides a remedy for enhancing PRO comprehension among medical students, but also aligns with the broader shift towards patient-centric, evidence-based healthcare practices.

### Electronic supplementary material

Below is the link to the electronic supplementary material.

Supplementary Material 1

## Data Availability

The data generated and analyzed during the current study are not publicly available as participants did not provide consent for data sharing, however de-identified data is available from the corresponding author on reasonable request.

## Abbreviations

PRO: Patient-reported outcome
LCME: Liaison Committee on Medical Education
ACGME: Accreditation Council for Graduate Medical Education
CMS: Centers for Medicare and Medicaid Services
