# Supplementary material for: Evaluating medical students’ knowledge of patient-reported outcomes and the impact of curriculum intervention in consecutive cohorts
Source: J Patient Rep Outcomes. 2023 Dec 13;7:131. doi: 10.1186/s41687-023-00670-z (PMC10719162; doi:10.1186/s41687-023-00670-z)
Supplement: Supplementary file 1 — Supplementary Material 1 [file 41687_2023_670_MOESM1_ESM.docx]

1. Do you know what a Patient Reported Outcome is?
   - 1. Yes
     2. No
2. Have you ever received formal education (e.g. a lecture) on Patient Reported Outcomes?
   - 1. Yes
     2. No
3. Have you ever used a Patient Reported Outcome (as a student, employee, or researcher)?
   - 1. Yes
     2. No
4. While on clerkship, have you ever seen a provider use a Patient Reported Outcome in patient care?
   - 1. Yes
     2. No
     3. I have not been on a clerkship
5. Which ONE of the following BEST describes the difference between a Patient Reported Outcome and other outcomes obtained in the office or hospital setting?
   - 1. They are not different.
     2. Patient reported outcomes measure safety, mortality, and efficiency of care while other outcomes measure efficacy.
     3. Patient reported outcomes measure efficacy of care, while other outcomes measure safety, mortality, and efficiency of care.
     4. **Patient reported outcomes are a measure of health that comes directly from the patient without interpretation by a clinician.**
     5. Patient reported outcomes are a measure of health that comes directly from the patient with interpretation by a clinician.
     6. I do not know.
6. A 73-year-old female with low back pain is seen in the physician’s office. She appropriately completed her health history form (past medical problems and surgeries) last night from the patient portal of the electronic record as well as entered in answers to questions about how she is feeling and functioning. At the office, the clinical technician obtained her blood pressure, asked quality questions, including if she has fallen in the past month, as well as if she lives alone and if she feels safe with her partner at home (domestic violence questions), and records them in the electronic record. She is led to the clinic room and her doctor asks her about her low back pain history, severity, and examines the patient’s lumbar spine for stability, motion, and strength. Lumbar spine x-rays are obtained and reviewed. The doctor summarizes the findings and discusses treatment options with the patient and together they make plans regarding the next steps in treatment.

Which of these are considered Patient Reported Outcomes? (Select all correct answers)

- 1. Health history form
  2. **Questions on feeling and function**
  3. Blood pressure value
  4. Quality questions on fall risk and domestic violence
  5. Reporting on low back pain history
  6. Pain
  7. Lumbar physical exam range of motion
  8. X-rays
  9. Shared decision-making discussion
  10. I do not know

1. A 69-year-old male with grade 3 (moderate) right knee arthritis sees his primary care physician to discuss total knee arthroplasty (TKA). He has no other medical conditions. He completes a validated Patient Reported Outcome measure assessing his current state of physical function which suggests he already functions very well, and it is very unlikely that a total knee replacement surgery will clinically improve his function.

What is the MOST likely appropriate next action for the physician?

- - 1. Refer the patient to an orthopedic surgeon for further evaluation.
    2. There is no need to incur further costs on the patient if they are functioning well.
    3. **Share and discuss the results with the patient.**
    4. Verify the patient’s answers with them.
    5. I do not know.

1. The Centers for Medicare & Medicaid Services (CMS) launched the comprehensive Meaningful Measures Initiative in 2017, which identifies high priority areas for quality measurement and improvement to improve patient and provider outcomes.

This initiative highlights the need to utilize Patient Reported Outcome measures in order to:

- - 1. Provide feedback to the providers
    2. Focus on major domain outcomes
    3. **Include the patient’s voice within the healthcare record without interpretation from others**
    4. Use measures that will advance innovative payment structures
    5. Accelerating the move to fully digital measures
    6. All of the above
    7. I do not know

1. Which of the following are MOST likely necessary for incorporating Patient Reported Outcomes into daily practice? (Select all that apply):
   - 1. **Not costly**
     2. **Minimal patient response burden**
     3. **Validated measures**
     4. Expensive
     5. Specific to each patient
     6. I do not know
2. True or False: Patient Reported Outcomes are intended to be completed by the respondent without help from anyone else, but if respondents are unable to answer on their own, may have someone else (“proxy”) report on their behalf.
   - 1. **True**
     2. False
     3. I do not know
3. True or False: It is the right of patients who complete Patient Reported Outcome measures to obtain the results.
   - 1. **True**
     2. False
     3. I do not know

For the following questions, select to what extent you agree or disagree with the statement below:

1. Patient Reported Outcomes are a key component in delivering high-quality care.
   - 1. Strongly agree
     2. Agree
     3. Neither agree nor disagree
     4. Disagree
     5. Strongly disagree
2. I feel prepared to use Patient Reported Outcomes in patient care:
   - 1. Strongly agree
     2. Agree
     3. Neither agree nor disagree
     4. Disagree
     5. Strongly disagree
3. I would use Patient Reported Outcomes in my future practice.
   - 1. Strongly agree
     2. Agree
     3. Neither agree nor disagree
     4. Disagree
     5. Strongly disagree
4. I am interested in learning more about Patient Reported Outcomes.
   - 1. Strongly agree
     2. Agree
     3. Neither agree nor disagree
     4. Disagree
     5. Strongly disagree
5. What is your current medical school training year?
   - 1. 1^st^ year
     2. 2^nd^ year
     3. 3^rd^ year
     4. 4^th^ year
     5. Other (MD/PhD)
6. How many months of clinical rotation training in medical school have you had to date?
   - 1. None
     2. Less than 3 months
     3. 3 to 6 months
     4. Between 6 to 12 months
     5. Greater than 12 months
7. What is your current primary career specialty interest?
   - 1. Anesthesiology
     2. Dermatology
     3. Emergency Medicine
     4. Family Medicine
     5. Internal Medicine
     6. Neurology
     7. Obstetrics and Gynecology
     8. Ophthalmology
     9. Pathology
     10. Pediatrics
     11. Psychiatry
     12. Radiation Oncology
     13. Radiology
     14. Surgery/Surgical subspecialty
     15. Undecided
     16. Other
8. With what gender do you identify? (select all that apply)
   - 1. Female
     2. Male
     3. Non-binary/third gender
     4. Transgender female
     5. Transgender male
     6. Prefer not to say
     7. Other
9. Which of the following best describes you? (select all that apply)
   - 1. American Indian or Alaska Native
     2. Asian
     3. Black or African American
     4. Hispanic or Latinx
     5. Native Hawaiian or Pacific Islander
     6. White
     7. Prefer not to say
     8. Other
